# Usefulness of measurement of heart rate variability by holter ECG in hemodialysis patients

**DOI:** 10.1186/s12882-016-0423-3

**Published:** 2017-01-05

**Authors:** Nanami Kida, Yoshiharu Tsubakihara, Hirota Kida, Shunro Ageta, Makoto Arai, Yoshinosuke Hamada, Nariaki Matsuura

**Affiliations:** 1 Department of Molecular Pathology, Osaka University Graduate School of Medicine and Health Science, 1-1 yamadaoka, 565-0871 Suita City, Osaka Japan; 2 Department of Blood Purification Center, Nagahara Hospital, 4-3-13 nagatanishi, 577-0016 Higashiosaka City, Osaka Japan; 3 Department of Management in Health Care Sciences, Graduate School of Health Care Science, Jikei Institute, 1-2-8 miyahara yodogawaku, 532-0003 Osaka City, Japan; 4 Department of Clinical Engineering, Osaka General medical Center, 3-1-56 bandaihigashi sumiyoshiku, 558-0056 Osaka City, Japan; 5 Department of Internal Medicine, Yuseikai Clinic, 10-39 hideincyo tennojiku, 543-0055 Osaka City, Japan

**Keywords:** Heart rate variability, Major adverse cardiac and cerebrovascular events, Hemodialysis patients

## Abstract

**Background:**

Major adverse cardiac and cerebrovascular event (MACCE) is one of most common complications of hemodialysis patients. Heart rate variability (HRV) is the predictor of death in heart disease patients. However, there are no studies on the role of HRV in hemodialysis patients.

**Methods:**

From September 2009 to March 2011, 24-h electrocardiography was performed in 101 hemodialysis patients. Standard deviation of sequential 5-minute N-N interval means (SDANN) and standard deviation of the N-N interval (SDNN) was examined by a 24-h ECG analysis. Patients were observed prospectively. The primary endpoints were incidence of MACCE and MACCE-free survival.

**Results:**

We studied 90 hemodialysis patients (64 males, 63.4 ± 11.8 years old). During a follow-up period of 32.0 ± 11.7 months, 33 patients developed MACCE. 24-h ECG showed mean SDNN 93.4 ± 33.4 ms and mean SDANN 83.2 ± 31.3 ms. MACCE group showed significantly lower SDNN and SDANN than event-free group. In Kaplan-Meier analysis higher SDNN and SDANN group showed significantly higher event-free survival rate than lower group. Using a Cox proportional hazards model, SDNN was independent prognostic factor while SDANN or diabetic status was not significant. In diabetic cases, there were no differences in any factors for the incidence of MACCE between higher SDNN, SDANN groups and lower groups. On the other hand in non-diabetic cases, lower SDNN or SDANN group developed significantly higher MACCE than higher groups.

**Conclusion:**

Measurement of HRV by Holter ECG is useful to predict MACCE in hemodialysis patients, especially non-diabetic group.

## Background

Patients on hemodialysis frequently have major adverse cardiac and cerebrovascular events (MACCE) which sometimes lead them to death [1, 2] and prediction and prevention of MACCE are important for them. Numerous factors have been reported to induce MACCE in hemodialysis patients. Chronic overhydration status in hemodialysis patients may induce structural or functional disorders in myocardium, leading to arrhythmia. Electrolyte imbalance and autonomic nerve disorder, sometimes observed in hemodialysis patients, may cause cardiac sudden death. Oxidative stress, inflammation and abnormal calcium or phosphate metabolism, which are specific to hemodialysis patients, may play some roles in atherosclerosis of coronary artery [3–5]. Hypertension, frequently found in hemodialysis patients, is reported to be the most important factor to cerebrovascular disorders [6]. Although detection of high risk group in hemodialysis patients who would develop MACCE is an important issue, there are no reports on appropriate prognostic biomarkers available for MACCE in hemodialysis patients [7, 8]. Here we propose the usefulness of Holter electrocardiogram (ECG) to predict MACCE in hemodialysis patients.

Holter ECG, conducted over a 24-h period, is widely used as non-invasive measures for the detection of cardiac arrhythmia which is not found in usual ECG. Holter ECG can also evaluate heartbeat change, such as heart rate variability (HRV), by measuring palmic fluctuation. HRV is an index of autonomic activity and includes the standard deviation of the N-N interval (SDNN) and standard deviation of sequential 5-minute N-N interval means (SDANN). SDNN estimates the standard deviation of the N-N intervals over 24 h, while SDANN reflects the standard deviation of the mean N-N intervals calculated in 5-min segments over 24-h. The N-N interval shows the R-R interval between consecutive QRS complex peaks during normal sinus rhythm. Larger fluctuation in the R-R intervals, thus higher HRV, can be found at rest in healthy individuals since heart beats can be physiologically changed by the influence of sympathetic and parasympathetic function according to circadian rhythm. On the other hand, R-R intervals become relatively stable and lower HRV is detected under strong stress or autonomic nerve dysfunction.

HRV is greatly influenced by age and heart rate [9]. Furthermore previous study suggested that patients with diabetes or hypertrophic cardiomyopathy showed decreased HRV [9–13]. It is also reported that the cardiac patients with decreased HRV showed poor prognosis [14, 15]. Lower HRV is reported to be associated with sudden cardiac death from cardiac arrhythmia or post myocardial infarction [16, 17]. In hemodialysis patients HRV is reduced and decreased HRV is observed already at the stage of chronic kidney failure prior to initiation of dialysis [18, 19]. However there are no studies on the role of HRV in the prognosis of hemodialysis patients. In this study, we examined the association between HRV and MACCE in hemodialysis patients to determine prognostic factors and the influence of diabetic status on HRV was also studied.

## Methods

From September 2009 to March 2011, 24-h ECG was performed in 101 patients on hemodialysis treated at Osaka General Medical Center, Yuseikai Clinic and Nagahara Hospital. Holter monitor (FM8800; Fukuda Denshi Co Ltd, Tokyo, Japan), was attached to the patients before the start of hemodialysis and data were collected on 2 channels for 24 h. SDANN and SDNN as an index of HRV were examined as well as the check of atrial and ventricular arrhythmias by a 24-h electrocardiographic analysis using an SCM-6600 System (Fukuda Denshi Co, Ltd, Tokyo, Japan).

Clinical data were utilized, including age, gender, body mass index (BMI), duration of hemodialysis, medication of antihypertensives, blood pressure, and fluid removal volume. Biochemical analysis was performed before and after hemodialysis, including serum sodium, potassium, calcium, chloride, blood urea nitrogen, creatinine, albumin, and hemoglobin levels. All the patients did not undergo hemodiafiltration and acetate free biofiltration, but underwent hemodialysis with bicarbonate dialysate and high-flux polysulfone, ethylene vinyl alcohol, and polyethersulfone membrane with a surface area ranging from 0.8 to 2.5 m^2^.

The hemodialysis patients were examined prospectively for observational study until December 31, 2013. The primary endpoints were incidence of MACCE and MECCE-free survival. The patients were divided into low-level (SDNN 45 cases, SDANN 44 cases) and high-level groups (SDNN 45 cases, SDANN 46 cases) respectively according to the median value for cutoff line, and all-cause death and MACCE during the observation period were checked.

Subjective 101 patients included 36 females and 65 males. One patient with chronic atrial fibrillation was also excluded because HRV could not be obtained. Ten patients who died of cancer or complication after renal transplantation during the observation period were excluded from the study. Therefore 90 patients (26 females and 64 males) were studied during the observation period.

Informed consent was obtained from all study participants in accordance with the Helsinki Declaration, and the study was approved by the ethical review boards of the treating institutions. Data are shown as mean ± standard deviation (SD) and statistical analysis was conducted using IBM SPSS software (IBM SPSS version 11.0; IBM, Chicago, Illinois, USA). Continuous variables were compared using a paired t-test, and data of the nominal scale were analyzed by the χ2 test. Event-free survival data are depicted in a Kaplan-Meier analysis. Univariate and multivariate analyses were conducted to determine the risk factors. Significance level for the primary endpoint was set at 5%.

## Results

Ninety patients (26 females and 64 males) were studied during the observation period; baseline clinical characteristics and biochemical data are shown in Table 1. Mean age was 63.4 ± 11.8 years, with mean 68.4 ± 73.4 months duration on hemodialysis. For primary cause of hemodialysis, 44 patients (48.9%) had diabetic nephropathy, 30 patients (33.3%) had chronic glomerulonephritis, and 16 (17.8%) had unknown or other diseases. Mean BMI was 21.6 ± 3.2 kg/m2. Forty-four patients were treated with angiotensin receptor blockers, 39 with beta blockers, 48 with calcium channel blockers, and 4 with angiotensin-converting enzyme inhibitors. The mean volume of water removed during hemodialysis of 3–4 h was 2.2 ± 1.2 kg and the mean systolic/diastolic blood pressure prior to the hemodialysis was 153.7 ± 19.1 mmHg/122.3 ± 18.2 mmHg. Blood biochemistry data before hemodialysis were as follows: hemoglobin (Hb), 10.1 ± 1.1 g/dl; albumin, 3.9 ± 0.5 g/dl; serum sodium, 138.3 ± 3.5 mEq/l; and serum potassium, 4.3 ± 0.7 mEq/l.

**Table 1:** Characteristics of the study subjects

All patients (*n* = 90)	
Age (year)	63.4 ± 11.8
Gender (f/m)	26:64
Duration of HD (months)	68.4 ± 73.4 (1–328)
Primary Cause of HD, n (%)	
Diabetic Nephropathy	44 (48.9%)
Chronic glomerulonephritics	30 (33.3%)
Unknown or others	16 (17.8%)
Body Mass Index (kg/m2)	21.6 ± 3.2
PVC,n (%)	48 (53.3%)
Medication, n (%)	
ARB	54 (60.0%)
Beta blockers	39 (43.3%)
48 (53.3%)	Ca channel blockers
ACE	4 (4.4%)
Hemoglobin (g/dl)	10.1 ± 1.1
Albumin (g/dl)	3.9 ± 0.5
pre/postHD Na (mEq/l)	138.3 ± 3.5/139.9 ± 2.1
pre/postHD K (mEq/l)	4.3 ± 0.7/3.3 ± 0.4
pre/postHD Ca (mg/dl)	9.2 ± 1.3/9.5 ± 1.0
Fluid removal (kg)	2.2 ± 1.2
Pre/post HD systolic blood pressure (mmHg)	153.7 ± 19.1/122.3 ± 18.2
Pre/post HD diastolic blood pressure (mmHg)	70.4 ± 14.3/73.1 ± 16.6

*PVC* premature ventricular contraction, *ARB* angiotensin receptor blocker, *Ca channel blockers* calciumchannel blockers, *ACE* angiotensin-converting enzyme inhibitor

Twenty-four hour ECG shows mean N-N 805.4 ± 107.2 ms; mean SDNN 93.4 ± 33.4 ms (median 91.5) and mean SDANN, 83.2 ± 31.3 ms (median 79.3). The patients were divided into low-level (SDNN 45 cases, SDANN 44 cases) and high-level groups (SDNN 45 cases, SDANN 46 cases) respectively according to the median value for cutoff line, and all-cause death and MACCE during the observation period were checked. During 24-h Holter ECG 48 patients developed premature ventricular contraction (PVC); 41 cases with low-risk PVCs and 7 cases with high-risk ones such as couplet PVC and PVC short run.

For the mean observation period of 32.0 ± 11.7 months (range: 1–49 months), 33 patients (36.7%) developed MACCE. Thirteen patients (39.4%) died during the observation period; cause of death is heart failure in 10 patients, arrhythmia in 2 patients and cerebral bleeding in 1 patient. Seventeen (51.4%) and 4 (12.1%) patients developed cardiovascular and cerebrovascular complications respectively.

There were no statistical differences in age, hemodialysis duration, BMI, fluid removal volume, Hb, albumin, electrolytes and administration of antihypertensives between the MACCE group and non-MACCE group. The systolic blood pressure change between pre- and post-hemodialysis was higher in event group than event-free group (35.3+/−19.9 vs 33.0+/−18.6 mmHg; *p* < 0.05). Event group included more diabetes mellitus (DM) patients than event-free group (66.7 vs 22.2%; *p* < 0.01). Event group showed much lower SDNN and SDANN than event-free group (SDNN 78.3+/−31.2 vs 102.1+/−31.7 ms; *p* = 0.00, SDANN 70.7+/−30.7 vs 90.2+/−29.5 ms; *p* = 0.00) (Table 2).

**Table 2:** Correlated factors with incidence of MACCE

	With MACCE (*n* = 33)	Without MACCE (*n* = 57)	*P* value
Age (year)	63.7 ± 11.9	63.3 ± 11.8	0.87
HD duration (mounts)	64.7 ± 68.9	70.9 ± 76.1	0.69
DM (n,%)	22 (66.7)	11 (33.3)	0.01
BMI (kg/m2)	21.3 ± 3.5	21.8 ± 3.0	0.49
PVC (n,%)	26 (78.8)	7 (21.2)	0.01
Hb (g/dl)	10.4 ± 1.0	10.5 ± 1.2	0.78
Alb (g/dl)	3.8 ± 0.4	3.9 ± 0.5	0.50
Fluid removal (kg)	2.3 ± 1.3	2.2 ± 1.2	0.51
ARB (n,%)	16 (48.5)	23 (40.4)	0.45
β blocker (n,%)	19 (57.6)	35 (61.4)	0.72
Ca channel blockers (n,%)	17 (51.5)	31 (54.3)	0.63
Na (mEq/l)	139 ± 3.4	138.2 ± 3.5	0.30
K (mEq/l)	4.4 ± 0.8	4.2 ± 0.7	0.47
Ca (mEq/l)	9.1 ± 1.3	9.3 ± 1.3	0.49
SDNN (ms)	78.3 ± 31.2	102.1 ± 31.7	<0.001
SDANN (ms)	70.7 ± 30.7	90.2 ± 29.5	<0.001
⊿Sys blood pressure (mmHg)	35.3 ± 19.9	33.0 ± 18.6	0.05
⊿Dia blood pressure (mmHg)	14.5 ± 7.8	13.2 ± 8.1	0.47

*DM* diabetes mellitus, *BMI* body mass index, *PVC* premature ventricular contraction, *Hb* hemoglobin, *Alb* albumin, *ARB* angiotensin receptor blocker, *Ca channel blockers* calciumchannel blockers, *SDNN* standard deviation of the NN interval, *SDANN* standard deviation of sequential five-minute N-N interval means

When we compare low and high SDNN groups in the clinical and biochemical data, there were no statistical differences in age, hemodialysis duration, BMI, administration of antihypertensives, Hb, albumin, fluid removal, blood pressure and blood pressure change except DM; 29 DM cases (55.1%) in 49 low SDNN patients vs 15 DM cases (36.6%) in 41 high SDNN ones ( *p* < 0.05). However, incidence of MACCE in low SDNN group was 55.1% (27/49), much higher than that in high SDNN group, 14.6% (6/41) (*p* < 0.01). Also low and high SDANN groups were compared in the similar ways. There were no statistical differences in any data including DM. However, low SDANN group had much higher incidence of MACCE than high SDANN group (47.4% (27/57) vs 18.2% (6/33); *p* = 0.01). Low SDNN group developed PVC more frequently than high SDNN group during 24-h ECG (*p* < 0.05) as well as more frequent incidence of PVC in low SDANN group (*p* < 0.03).

**Fig. 1:**
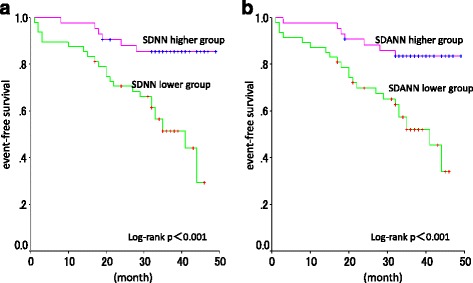
Event-free survival of hemodialysis patients with higher and lower heart rate variability. captions: **a** SDNN higher group showed significantly higher MACCE-free survival than SDNN lower group. **b** SDANN higher group showed significantly higher MACCE-free survival than SDANN lower group

In Kaplan-Meier analysis higher SDNN group showed much higher event-free survival rate than lower SDNN group (Log rank, *p* = 0.00) as well as higher SDANN group showed significantly higher survival rate (Log rank, *p* = 0.01) (Fig. 1). In the longer follow-up time the difference of event-free survival between SDNN higher and lower groups, as well as SDANN higher and lower groups, seems to be increased. In the analysis using a Cox proportional hazards model, SDNN was determined to be an independent prognostic factor (*p* = 0.02; hazard ratio: 0.57) while SDANN or diabetic status was not significant in multivariate analysis (Table 3).

**Table 3:** COX regression model for MACCE

	Univariate analysis		Multivariate analysis	
	*p* value	Hazard ratio (95% CI)	*p* value	Hazard ratio (95% CI)
Age	0.78	1.0 (0.98–1.02)		
HD duration	0.84	1.0 (0.99–1.00)		
DM	0.02	1.6 (1.09–2.56)	0.06	1.5 (0.9–2.4)
SDNN	0.01	0.5 (0.37–0.87)	0.02	0.57 (0.36–0.89)
SDANN	0.12	0.7 (0.48–1.14)		
PVC	0.06	2.1 (0.88–2.73)		
ARB	1.00	1 (0.50–1.99)		
β blocker	0.54	0.9 (0.57‐1.34)		
Hb	0.90	1.0 (0.82–1.20)		
Alb	0.11	1.5 (0.91–2.46)		
Fluid removal	0.30	1.1 (0.92–1.32)		
⊿Systolic Blood Pressure	0.20	1.0 (0.99–1.02)		

*DM* diabetes mellitus, *SDNN* standard deviation of the NN interval, *SDANN* standard deviation of sequential five-minute N-N interval means, *PVC* premature ventricular contraction, *ARB* angiotensin receptor blocker, *Hb* hemoglobin, *Alb* albumin

Predictive factors for MACCE are analyzed separately in both diabetic and non-diabetic patients. In diabetic 44 cases 22 patients (50%) developed MACCE and there were no significant differences in any factors including SDNN and SDANN for the incidence of MACCE. On the other hand 11 patients (23.9%) developed MACCE in non-diabetic 46 cases, much less rate than diabetic group. There were no significant differences in age, hemodialysis duration, hemoglobin level, albumin level, or BMI for MACCE. However, lower SDNN or SDANN group developed significantly higher MACCE than higher groups (*p* = 0.00 or *p* = 0.04, Table 4).

**Table 4:** MACCE-correlated factors in diabetic and non-diabetic subjects

DM (*n* = 44)				Non-DM (*n* = 46)		
	With MACCE (*n* = 22)	Without MACCE (*n* = 22)	*p* value	With MACCE (*n* = 11)	Without MACCE (*n* = 35)	*p* value
Age (year)	61.9 ± 12.8	63.1 ± 12.6	0.75	67.4 ± 9.6	63.4 ± 11.4	0.41
HD duration (months)	46.0 ± 11.6	46.6 ± 10.5	0.97	102.1 ± 81.8	86.3 ± 86.1	0.83
Alb (g/dl)	3.9 ± 0.4	4.0 ± 0.5	0.60	3.7 ± 0.4	3.9 ± 0.5	0.86
Hb (g/dl)	10.5 ± 1.1	10.7 ± 1.0	0.61	10.3 ± 0.8	10.4 ± 1.3	0.81
Fluid removal (kg)	2.4 ± 1.5	2.1 ± 1.1	0.41	2.2 ± 1.1	2.2 ± 1.2	0.91
SDNN (Low,n%)	16 (72.7%)	11 (50.0%)	0.12	7 (63.6%)	11 (31.4%)	0.05
SDANN (Low,n%)	16 (72.7%)	13 (59.1%)	0.34	8 (72.7%)	9 (25.7%)	0.01

*Alb* albumin, *Hb* hemoglobin, *SDNN* standard deviation of the NN interval, *SDANN* standard deviation of sequential five-minute N-N interval means

## Discussion

Although the prognosis of the hemodialysis patients has been improved, they still have higher risk for MACCE and earlier detection of MACCE is important in clinical practice [20]. In hemodialysis facility anemia, nutrition and bone metabolic markers are usually checked by the laboratory examination, but it is difficult for the prediction of MACCE from those laboratory data. Radiography like coronary angio-CT or ultrasonography is not usually so convenient in hemodialysis facility and sufficient examination for early detection is not performed. In this study we investigate the possibility of prediction of prognosis of the hemodialysis patients by Holter ECG, easy and non-invasive measures available in usual hemodialysis facility.

HRV obtained by Holter ECG reflects the balance of sympathetic and parasympathetic nervous function [9, 10]. Decreased HRV defined by SDNN or SDANN, which shows no difference in heartbeats between in the daytime and at night, is considered as a state of sympathetic hyperactivity. Activated condition of sympathetic nerve function is known to induce lethal arrhythmia leading to sudden death [21]. An earlier study reported that prognosis of patients with heart failure or ischemic heart disease was intimately linked to HRV [22, 23].

In this study the incidence of MACCE was significantly correlated with diabetic status, low SDNN, low SDANN and high blood pressure change during hemodialysis. Our result is consistent with the previous reports in which the hemodialysis patients from diabetic nephropathy have higher risk for MACCE [24]. Our results that the patients with MACCE showed significantly lower SDNN and SDANN were noteworthy because decreased HRV might reflect high cardiac overload resulting in MACCE. Higher blood pressure change during hemodialysis significantly associated with MACCE means that cardiac function was so poor that decreased circulating blood volume due to hemodialysis may develop higher blood pressure change.

In this study SDNN and SDANN low-level groups had higher incidence of MACCE and worse outcome than the high-level groups and also SDNN and SDANN lower groups showed significantly lower event-free survival in Kaplan-Meier analysis than higher groups. Furthermore SDNN was an independent prognostic factor in COX-hazard analysis while SDANN was not. SDNN estimates the standard deviation of the mean values of NN interval for 24 h and reflects an HRV change over the long-term more strongly compared with SDANN which indicates the standard deviation of the mean N-N intervals calculated in 5-min segments over 24-h. Therefore, SDNN is a more sensitive predictor of prognosis than SDANN. These results suggest that body fluid volume should be strictly regulated by hemodialysis and that early detection of cardiovascular complications should be recommended in the hemodialysis patients with HRV decrease since those patients were at high risk for MACCE. When we analyzed incidence of PVC, low SDNN group developed PVC more frequently as well as low SDANN group. PVC has been reported to be associated with age, sympathetic nerve imbalance and cardiac dysfunction. Furthermore, the patients with PVC had more incidence of MACCE (*p* < 0.01), suggesting low SDNN and SDANN might result ventricular arryhtmia and that could be predictable factors for MACCE .

In the current study HRV was not correlated with age or dialysis history. Our result might suggest that some patients showed activated sympathetic function resulting in poor prognosis already at the early stage of chronic renal failure. Generally long term dialysis is supposed to induce MACCE. However, there are high risk group of hemodialysis patients even at the initiation stage of hemodialysis with the increased older population or diabetic status for hemodialysis. As previous study showed HRV are already decreased at the stage of advanced chronic kidney disease prior to initiation of dialysis, the measurement of HRV at the initiation of hemodialysis would be useful for the detection of high risk group of MACCE [18]. In this study there were no differences in SDNN and SDANN between no medications and medications with of beta blocker or calcium blocker. Previous study reported medication with calcium blocker induced activation of parasympathetic function, resulting in short-term HRV such as mean R-R intervals calculated in 5-min segments [25]. In this study we tested only long-term HRV and so no differences were observed.

The hemodialysis patients with diabetic nephropathy as primary disease have been reported to show poor prognosis. In this study low SDNN or SDANN group showed similar incidence of MACCE to high SDNN or SDANN group in the diabetic patients, whereas low SDDN or SDANN group demonstrated significantly higher incidence of MACCE than high group in non-diabetic patients. Earlier research has shown that diabetic condition itself is a strong risk factor for MACCE and the contribution of HRV might be relatively low. On the other hand, low SDNN or SDANN patients show higher incidence for MACCE in non-diabetic patients in our study, suggesting low HRV might be a strong risk factor for prognosis in non-diabetic hemodialysis patients.

There are several limitations to our study. First, the sample size of 90 patients was small. Secondly cardiac function by echocardiography was not evaluated since echocardiogram was not equipped in one clinic and the association of SDNN and SDANN with cardiac function such as left ventricular end-diastolic dimension and left ventricular ejection fraction. Thus the relationship between MACCE and cardiac function was not discussed sufficiently.

## Conclusion

Measurement of HRV by Holter ECG is useful to predict MACCE in hemodialysis patients, especially non-diabetic group.

## Abbreviation

BMI: Body mass index
ECG: Electrocardiogram
Hb: Hemoglobin
HRV: Heart rate variability
MACCE: Major adverse cardiac and cerebrovascular events
PVC: Premature ventricular contraction
SDANN: Standard deviation of sequential 5-minute N-N interval means
SDNN: Standard deviation of the N-N interval
